# Preoperative Risk Factors for Opioid Use Predict Self‐Reported Health and Function Up to 6 Months After Anterior Cruciate Ligament Reconstruction

**DOI:** 10.1002/ars2.70066

**Published:** 2026-09-06

**Authors:** Eric V. Neufeld, William DeGouveia, Camille Pinpin, Matthew Zinner, Michael J. Sayegh, Michael J. Meade, Steven E. Rokito

**Affiliations:** ^1^ Northwell New Hyde Park New York U.S.A.; ^2^ Department of Orthopaedic Surgery Long Island Jewish Medical Center New Hyde Park New York U.S.A.

## Abstract

**Purpose:**

To identify risk factors that predict the need for rescue opioid use and patient‐reported outcomes following an opioid‐limiting postoperative analgesia protocol after anterior cruciate ligament reconstruction.

**Methods:**

This prospective cohort study recruited 44 patients undergoing primary arthroscopic‐assisted anterior cruciate ligament reconstruction. Preoperatively, patients completed questionnaires including demographics, medical history, Visual Analog Scale (VAS), Brief Resilience Scale, Six‐Item State‐Trait Anxiety Inventory, Patient Health Questionnaire‐2, Single Assessment Numeric Evaluation score, and patient‐reported outcome measures (PROMs) including Patient‐Reported Outcomes Measurement Information System physical component. Postoperatively, patients received acetaminophen, meloxicam, tramadol, gabapentin, and oxycodone. Morphine milligram equivalents (MMEs) and VAS were assessed at multiple timepoints through 6 months. PROMs were completed at 3 and 6 months. Patients were analyzed as a single group and partitioned by Brief Resilience Scale and Six‐Item State‐Trait Anxiety Inventory scores into low‐normal (1.00‐4.30) and high (4.31‐5.00) resilience, and low (20‐37) and moderate‐high (38‐80) anxiety groups, respectively.

**Results:**

Prior opioid use positively predicted VAS and MME at 48 to 72 hours and MME at 6 weeks. Body mass index positively predicted MME at 2 weeks. Surgical history negatively predicted VAS at 48 to 72 hours, but positively predicted change in Single Assessment Numeric Evaluation score at 3 and 6 months and Patient‐Reported Outcomes Measurement Information System physical component score at 6 months.

**Conclusions:**

Only 4.8% of patients required opioid medications more than 2 weeks postoperatively. Patients with prior opioid use exhibited greater postoperative pain and narcotic consumption. Higher body mass index linked to increased opioid use at 2 weeks, whereas individuals with prior surgical history reported less pain and improved PROMs postoperatively. Measurements of patient resilience and anxiety show little effect on postoperative pain, opioid consumption, and PROMs requiring further study.

**Level of Evidence:**

Level III, retrospective prognostic case series.

## Graphical abstract

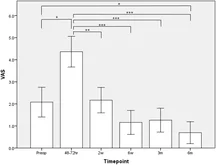

Prescription opioid abuse remains an ongoing public health crisis, particularly in patients undergoing orthopaedic surgery.^1^, ^2^ Orthopaedic surgery is the largest contributor to this epidemic, particularly in the postoperative setting, accounting for 8.8% of postoperative opioid dependence cases.^3^ As a result, optimization of postoperative pain management presents a powerful approach to ameliorate the opioid crisis.

Although opioid prescriptions are common in the postoperative period across multiple surgical disciplines, even short‐term exposure to postoperative narcotics has been shown to increase the risk of long‐term misuse, abuse, and addiction.^4^ The complete elimination of opioid prescriptions may lead to decreased satisfaction in some patients.^5^ However, opioid‐sparing multimodal pain regimens have shown effective pain control with a limited side effect profile after common orthopaedic sports medicine procedures. Therefore, it is of paramount importance to identify patient factors that can aid physicians in tailoring narcotic prescriptions to only those individuals who truly require them.

Preoperative opioid use is a well‐established predictor of poorer postoperative outcomes and pain control. In shoulder surgery patients, it has been independently associated with greater pain, reduced function, and lower social satisfaction.^6^ Okoli et al. found that preoperative opioid use was the strongest predictor of both filling a second postoperative opioid prescription and continued opioid use beyond 6 months after various orthopaedic procedures.^7^ Similarly, Singh et al. reported that preoperative opioid use in total hip arthroplasty was associated with indicators of hyperalgesia and poorer clinical outcomes.^8^ Despite extensive evidence across orthopaedic subspecialties, the specific impact of preoperative opioid use on outcomes after anterior cruciate ligament reconstruction (ACLR) remains underexplored and warrants further investigation.

Resilience, defined as the ability to recover from psychological or physical stress, has emerged as an important psychometric factor influencing outcomes such as quality of life, suicide risk in active‐duty military personnel, and postoperative recovery after orthopaedic procedures.^9^, ^10^ The Brief Resilience Scale (BRS), a 6‐item Likert scoring scale, is increasingly used to assess resilience in postoperative outcome studies. Higher preoperative BRS scores are associated with better physical and mental health outcomes after both total knee arthroplasty and total shoulder arthroplasty.^11^, ^12^, ^13^

The purposes of this study were to identify risk factors that predict the need for rescue opioid use and patient‐reported outcome measures (PROMs) following an opioid‐limiting postoperative analgesia protocol after ACLR. We hypothesized that prior opioid use, high anxiety, and low resilience would correlate with increased perception of pain and, therefore, narcotic use in the postoperative period.

## METHODS

This prospective cohort study evaluated patients undergoing primary arthroscopic‐assisted ACLR by a sports‐fellowship‐trained orthopaedic surgeon (S.E.R.) at a single academic health institution. This investigation was approved by the Institutional Review Board. Patients first underwent brief diagnostic knee arthroscopy in which native anterior cruciate ligament was debrided and tunnel sites prepped. Graft harvest followed, with technique varying by graft choice. Femoral and tibial tunnels were appropriately sized and drilled, followed by graft passage via shuttle sutures and fastening with interference screws.

Patient recruitment was performed by the attending physician at the first preoperative visit, and all participants meeting inclusion criteria provided informed consent. Inclusion criteria was defined as any patient over the age of 18 undergoing primary ACLR with either autograft or allograft. Patients undergoing revision ACLR and patients under the age of 18 were excluded from the study. A total of 44 patients were enrolled in the study. At the patient's preoperative visit, a member of the research staff administered questionnaire forms including demographics, Visual Analog Scale (VAS), BRS, Six‐Item State‐Trait Anxiety Inventory, Patient Health Questionnaire‐2, Single Assessment Numeric Evaluation, and various PROMs, including the Patient‐Reported Outcomes Measurement Information System physical component, International Knee Documentation Committee score, and Tegner scores. Patient's medical and surgical history including past opioid use, history of connective tissue disease, weight, and functional status, was also recorded. Further, all participants were educated on how to take the multimodal, opioid‐limiting pain regimen postoperatively in the setting of breakthrough pain not otherwise alleviated by nonopioid analgesics (Table 1).

**TABLE 1:** Postoperative Opioid‐Limiting Pain Cocktail

Medication	Directions
Acetaminophen 500 mg	2 tablets Q8H for 10 days
Gabapentin 100 mg	2 tablets Q8H for 5 days
Tramadol 50 mg	1 tablet Q8H for 5 days
Meloxicam 15 mg	1 tablet QD for 15 days
Oxycodone 5 mg	1 tablet for breakthrough pain only (10 tablets)

Q8H, every eight hours; QD, once per day.

Patients were followed over the course of 6 months and were queried via telephone calls and emails at multiple timepoints. At 48 to 72 hours, 2 weeks, and 6 weeks, patients were asked the cumulative number of rescue 5 mg oxycodone tablets they had taken which were recorded as morphine milligram equivalents (MMEs) using the conversion 5 mg = 7.5 MME. VAS was also assessed at these timepoints, as well as 3 and 6 months. Medication nonadherence was defined as missing a single dose of any acetaminophen, gabapentin, tramadol, or meloxicam. At 2 weeks, the following 2 questions were administered from the Hospital Consumer Assessment of Healthcare Provider and Systems to assess satisfaction with analgesia: (1) “In the time after surgery, how often was your pain well controlled?” and (2) “In the time after surgery, how often did hospital staff do everything they could to help you with your pain?” The answer choices were “Never,” “Sometimes,” “Usually,” or “Always.”

### Statistical Analysis

All patients were reviewed and analyzed as a single group and stratified by BRS score into low‐normal resilience (1.00‐4.30) and high‐resilience (4.31‐5.00) groups based on the cutoff values obtained from a previous study.^13^ Patients were also partitioned (independent of BRS score) by Six‐Item State‐Trait Anxiety Inventory score into low‐anxiety (20‐37) and moderate‐high anxiety (38‐80) cohorts.^14^ Statistical comparisons between timepoints for VAS, MME, and PROMs analysis were performed via multiple linear regression, Welch's t‐tests, Fisher's exact tests, 1‐way repeated‐measures analysis of variance followed by Tukey's post hoc tests; the family‐wise error rate (*α* = 0.05) was maintained via Bonferroni corrections. All analyses were conducted in Excel (Microsoft, Redmond, WA) and SPSS (version 27.0; IBM, Armonk, NY).

## RESULTS

### Participants

A total of 44 patients were initially enrolled with a mean (standard deviation) age of 26.6 (12.8) years (Table 2). Gender distribution was 28 (63.6%) men and 16 (36.4%) women. Mean body mass index (BMI) was 26.7 (5.4) kg/m^2^. Most ACLRs were performed using a bone–patellar tendon–bone autograft (68.2%) followed by bone–patellar tendon–bone allograft (15.9%), hamstring autograft (13.6%), and quadriceps autograft (2.3%). Thirty‐eight patients (86.3%) completed follow‐up at 3 months and 32 patients (72.7%) at 6 months. The medication adherence rate was 84.4% and 67.4% at 48 to 72 hours and 2 weeks, respectively. VAS score predictably peaked at 48 and 72 hours and then declined at subsequent timepoints (Figure 1). Cumulative opioid consumption averaged less than 1 oxycodone tablet (7.0 MME) at 48 to 72 hours and marginally increased to 9.7 MME by 6 weeks. Regarding satisfaction with analgesia (Hospital Consumer Assessment of Healthcare Provider and Systems question 1), 63.6% and 24.2% of patients reported that their pain was “Always” and “Usually” well controlled, respectively. Regarding care providers’ assistance with analgesia (Hospital Consumer Assessment of Healthcare Provider and Systems question 2), 90.9% and 3.0% of patients stated that they were “Always” and “Usually” satisfied, respectively.

**TABLE 2:** Patient Demographics and Comorbidities

Independent Variable	N (%)	Mean (SD)
Age (years)	‐	26.6 (12.8)
BMI	‐	26.7 (5.4)
BRS	‐	3.9 (0.7)
STAI‐6	‐	37.4 (13.6)
PHQ‐2	‐	0.7 (1.4)
Sex	16 women (36.4%)	‐
History of opioid use	13 (29.5%)	‐
Smoking	2 (4.5%)	‐
Illicit drug use	2 (4.5%)	‐
Alcoholism	0 (0%)	‐
Psychiatric disorder	1 (2.3%)	‐
History of prior surgery	12 (27.3)	‐
DM2	0 (0%)	‐
HTN	0 (0%)	‐
CAD	0 (0%)	‐

BMI, body mass index; BRS, Brief Resilience Scale; CAD, coronary artery disease; DM2, noninsulin‐dependent diabetes mellitus; HTN, hypertension; PHQ‐2, Patient Health Questionnaire‐2; SD, standard deviation; STAI‐6, Six‐Item State‐Trait Anxiety Inventory.

**FIGURE 1:**
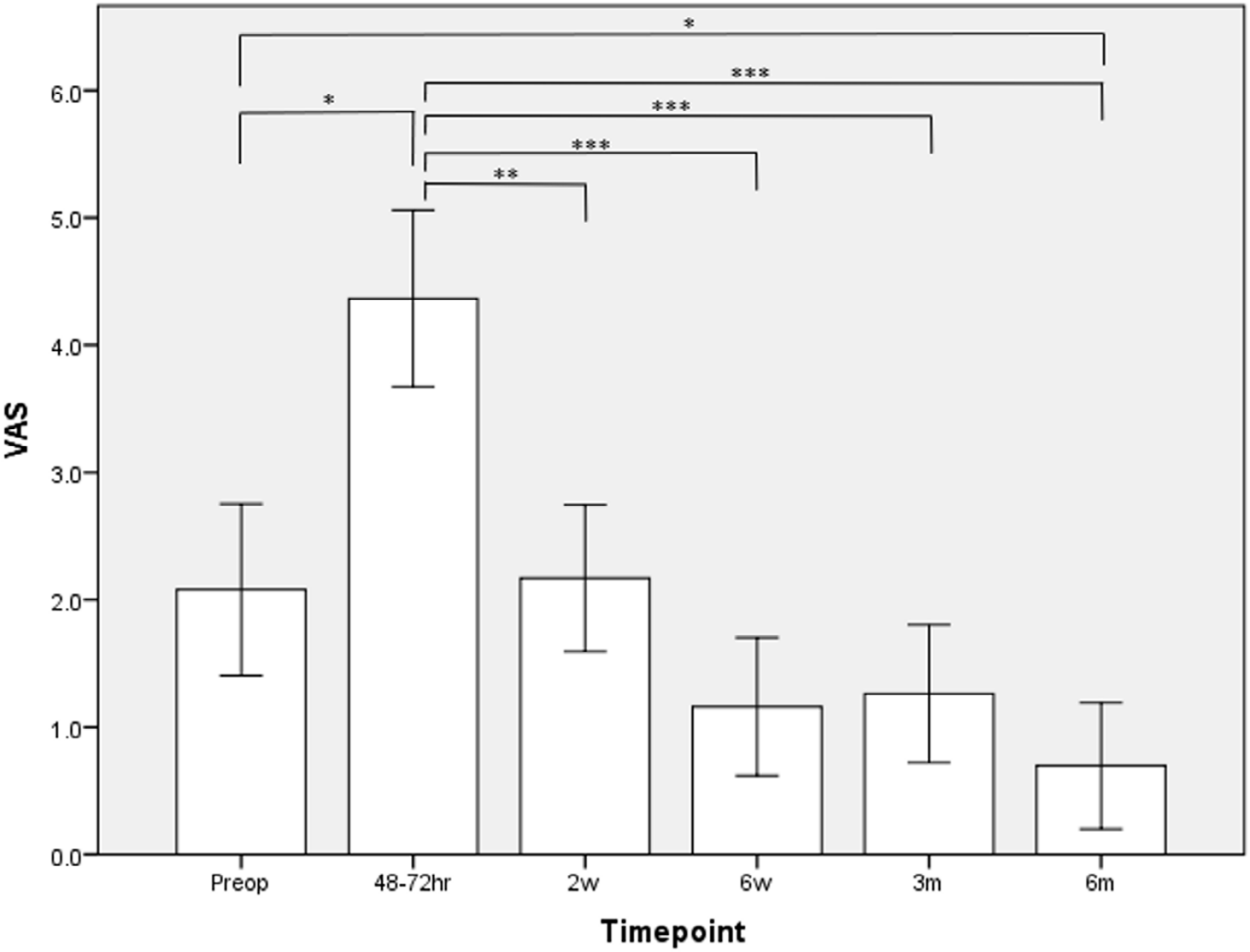
VAS scores at all timepoints. *P < .05, **P < .01, ***P < .001. (VAS, Visual Analog Scale.)

### Resilience and Anxiety

Compared with the low‐normal resilience group, the high‐resilience group was older (39.5 vs 23.8 years, *P* = .029) and showed lower Six‐Item State‐Trait Anxiety Inventory scores (27.5 vs 39.9, *P* = .002) but higher preoperative Patient‐Reported Outcomes Measurement Information System mental component scores (61.8 vs 52.4, *P* = .004). Although the moderate‐high anxiety cohort was younger (22.3 vs 31.0 years, *P* = .034), the low‐anxiety group showed higher BRS scores (4.1 vs 3.5, *P *= .005). There were no other significant differences in demographics or outcomes between either the low‐normal and high‐resilience groups or the low and moderate‐high anxiety cohorts.

### Predictors of Outcomes

A history of prior opioid use positively predicted VAS (*β* = 2.0; *P* = .024; standard error = 0.813; *R* ^2^ = 0.558) and MME at 48 to 72 hours (*β* = 16.9; *P* = .009; standard error = 6.017; *R* ^2^ = 0.447) (Table 3). It further positively predicted MME at 6 weeks (*β* = 24.0; *P* = .030; standard error =  10.518; *R* ^2^ = 0.334). Additionally, only 2/41 (4.8%) patients continued taking opioids after 2 weeks, both of whom had prior opioid use. BMI positively predicted MME consumption at 2 weeks (*β* = 2.0; *P* = .036; standard error = 0.885; *R* ^2^ = 0.472). The presence of surgical history negatively predicted VAS at 48 to 72 hours (*β* = −3.6; *P* < .001; standard error = 0.859; *R* ^2^ = 0.558). However, surgical history positively predicted change in Single Assessment Numeric Evaluation score at 3 months (*β* = 36.9; *P* = .017; standard error = 13.98; *R* ^2^ = 0.419) and 6 months (*β* = 35.4; *P* = .016; standard error = 12.406; *R* ^2^ = 0.615). It further positively predicted change in Preoperative 10‐Item Patient‐Reported Outcomes Measurement Information System physical component score at 6 months (*β* = 8.3; *P* = .021; standard error = 3.083; *R* ^2^ = 0.601).

**TABLE 3:** Predictors of Outcomes From Multiple Linear Regression

Timepoint	Predictor	Outcome	*β*	*P* Value
48‐72 hours	Prior opioid use	VAS	2.0	.024*
48‐72 hours	Prior opioid use	MME	16.9	.009**
48‐72 hours	Surgical history	VAS	‐3.6	<.001***
2 weeks	BMI	MME	2.0	.036*
6 weeks	Prior opioid use	MME	24.0	.030*
3 months	Surgical history	SANE	36.9	.017*
6 months	Surgical history	SANE	35.4	.016*
6 months	Surgical history	PROMIS‐10‐P	8.3	.021*

BMI, body mass index; MME, morphine milligram equivalent; PROMIS‐10‐P, Patient‐Reported Outcomes Measurement Information System physical component; SANE, Single Assessment Numeric Evaluation; VAS, Visual Analog Scale.

*Statistical significance at .05.

**Statistical significance at .01.

***Statistical significance at .001.

## DISCUSSION

In this study, we found that patients with previous opioid use reported higher postoperative VAS pain scores and required higher opioid pain medication compared with opioid naive patients. Additionally, only 2 patients continued taking opioids after 2 weeks postoperation, both of whom had a prior history of opioid use. These findings are in line with a study by Okoli et al. that evaluated 1384 patients undergoing common elective outpatient orthopaedic procedures, including ACLR, over a 1‐year period.^7^ They found that of the 20.8% of patients with a history of prior opioid use, 60.6% filled at least 1 additional opioid prescription postoperatively compared with 26.4% of opioid‐naive patients. Furthermore, of the opioid‐exposed patients, 29.3% continued to fill opioid prescriptions beyond 6 months postoperatively compared with only 6.0% of opioid‐naive patients. During their 12‐month postoperative period, opioid‐exposed patients filled significantly more opioid prescriptions and received significantly higher total amount in MMEs.

On top of narcotic consumption, the present study also linked prior opioid use to increased postoperative pain. This finding supports the investigation by Genel et al. who retrospectively assessed 1821 patients undergoing primary hip or knee replacement.^15^ They reported that patients with a history of prior opioid use had worse pain and function scores, as well as health quality of life at 6 months postoperatively. In addition to prior opioid use, the present investigation suggests that for every 1 kg/m^2^ increase in BMI, an additional 2.0 MME is consumed at 2 weeks postoperatively without an accompanying increase in pain. This phenomenon was also found by Lendrum et al. who reported that greater postoperative opioid use correlated with increased BMI in arthroplasty patients.^16^ Lustig et al. also supported our finding that higher BMI does not necessarily predict greater pain after ACLR.^14^

Results from the present study also showed a positive relation between prior surgical history and PROMs and decreased postoperative pain. In a Yang et al. review of 33 studies evaluating preoperative predictors of poor pain control, they showed that prior surgical history was not associated with increased odds of poor pain control.^17^ This study shows that previous surgery was not associated with poor pain control but was in fact a protective factor, leading to decreased postoperative pain and superior PROMS.

Although the present study determined that resilience and anxiety have little impact on postoperative pain, opioid consumption, and PROMs, the overall literature is more mixed. A study by Chavez et al. evaluated the role of BRS as a predictor of patient satisfaction with nonopioid pain management and PROMs after arthroscopic partial meniscectomy and chondroplasty.^18^ In comparing VAS pain scores of patients with low‐to‐normal resilience scores and those with high resilience scores at 0, 3, and 6 months, they found that preoperative resilience score does not correlate with postoperative patient‐reported functional outcome or satisfaction with a nonopioid pain regimen after knee arthroscopy. This conclusion echoes the one derived from the present study. In contrast, Armento et al. revealed that patients with higher preoperative grit levels showed superior PROMs and activity levels after ACLR. These findings suggest that more research is required to determine how the interplay of grit, resiliency, and anxiety affects patient's recovery from ACLR.^19^

Given the devastating effects of opioid misuse, there is a continued push to investigate the efficacy of alternative nonopioid analgesics. A recent prospective, randomized controlled trial by Jildeh et al. found that a multimodal nonopioid pain regimen provided equivalent pain control and patient outcomes after primary meniscus surgery while having an equivalent side effect profile.^20^ Furthermore, a randomized clinical trial by Swenson et al. found that patients receiving simultaneous doses of acetaminophen, celecoxib, and pregabalin had lower pain scores and decreased opioid consumption in outpatient ACLR.^21^ Further, Mengers et al. also revealed the efficacy of adjuvant non‐steroidal anti‐inflammatory drugs (NSAIDS) in the postoperative period in improving pain scores and diminishing postoperative opioid consumption.^22^ Despite these successes, some patients continue to require opioids to adequately manage their pain after ACLR. This study helps to illustrate which patients may be more likely to request opioids postoperatively, which provides valuable information to the orthopaedic surgeon who may be able to implement alternative strategies to help those high‐risk patients.

### Limitations

This investigation is not without limitations. A 6‐month follow‐up may not adequately capture the entire patient experience regarding postoperative pain as their recovery remained ongoing. Only 2 patients continued to use additional narcotics after 2 weeks; however, the possibility of new or continued use beyond 6 months cannot be ignored. Like all studies, this study suffered as patients were lost to follow‐up. Attrition by 6 months increases the likelihood of nonresponse bias and underpowered results. Furthermore, the heterogeneity in graft options across study participants may contribute to varying pain scores, PROMs, and opioid use. Another limitation is the lack of a control group. Although the decrease in pain and limited need for breakthrough narcotic use suggests the analgesia regimen was successful, there is no data comparing how many MMEs were consumed if the opioid‐limiting regimen was not prescribed at all. Lastly, our multimodal opioid‐limiting protocol also involved tramadol (equivalent to 0.1 MME), which is a mild‐to‐moderate opioid agonist but not factored into the opioid consumption calculations.

## CONCLUSIONS

Only 4.8% of patients required opioid medications more than 2 weeks postoperatively. Patients with prior opioid use exhibited greater postoperative pain and narcotic consumption. Higher BMI linked to increased opioid use at 2 weeks, whereas individuals with prior surgical history reported less pain and improved PROMs postoperatively. Measurements of patient resilience and anxiety show little effect on postoperative pain, opioid consumption, and PROMs requiring further study.

## DISCLOSURES

The authors (E.V.N., W.D., C.P., M.Z., M.J.S., M.J.M., S.E.R.) declare that they have no known competing financial interests or personal relationships that could have appeared to influence the work reported in this article.
